# Assessing Acceptability of Short Message Service Based Interventions towards Becoming Future Voluntary Blood Donors

**DOI:** 10.1155/2014/567697

**Published:** 2014-11-10

**Authors:** Sana Saleem, Anum Wasim, Sidra Sabih, Ayisha Farooq Khan, Madiha Hasan Rizvi, Umaima Ayesha Jillani, Mujtaba Jamal Syed, Madiha Mumtaz, Yasmeen Mumtaz, Abdul Moid Shehzad, Om Dawani, Saima Khan, Sheheryar Munir, Nava Asad, Abdul Nafey Kazi

**Affiliations:** ^1^Dow Medical College, Dow University of Health Sciences, Karachi 74200, Pakistan; ^2^Department of Community Medicine, Dow Medical College, Dow University of Health Sciences, Karachi 74200, Pakistan

## Abstract

All blood bank services, especially those of developing countries, face a major shortfall of blood donations due to lack of voluntary blood donors. Our study aims to evaluate the acceptability of Short Message Service based interventions towards becoming voluntary blood donors among medical university students of Karachi, Pakistan.* Methods*. A total of 350 medical students were approached in medical universities of Karachi, Pakistan, using a nonprobability convenient sampling technique. Data collectors administered a self-made questionnaire to each participant using an interview based format. All data was recorded and analyzed on SPSS 16.* Results*. 350 participants, having a mean age of 21.47 ± 1.36, were included in our study with 30.6% (107/350) being males and 69.4% (243/350) being females. 93.4% (327/350) of participants agreed that donating blood was healthy, but only 26% had donated blood in the past with 79.1% donating voluntarily. 65.7% (230/350) of the participants agreed to take part in Short Message Service based behavioral interventions to become voluntary blood donors with 69.7% (244/350) also agreeing that Short Message Service reminders will promote them to donate blood more often.* Conclusion*. With university students willing to become voluntary blood donors, Pakistani blood banks can carry out Short Message Service based interventions to encourage them to donate blood.

## 1. Introduction

The continuous major challenge faced by all blood services worldwide is the huge demand-supply gap between blood donors and patients [1]. Although blood donation forms an integral component of medical services, only 92 million blood donations were made to an estimated 234 million major operations performed in 2013 [2]. Developing countries, like Pakistan, experience an even greater crisis, owing to the high incidence of transfusion-requiring medical conditions like high risk obstetrics, thalassemia, trauma, and malaria.

In Pakistan, only 28 people donate blood for every population of 10000 [3]. Replacement donors and paying for donations contribute significantly to Pakistani blood banks that in turn promotes high risk collection of seropositive infectious blood as opposed to the low risk blood products obtained from voluntary donors [4]. In 2012, an estimated 70% donations were replacement or paid for donations with 50% cases receiving unscreened blood transfusions [5].

Recruitment of low risk voluntary blood donors is a primary solution of replenishing this shortfall in blood banks [6]. University student populations, medical university students in particular, are considered the best candidates to become voluntary blood donors. Nevertheless, experience at blood banks suggests that not all university students voluntarily donate blood on a regular basis [7]. The literature reveals misconceptions, lack of knowledge (even in medical students), and fear as the primary factors preventing students from becoming voluntary blood donors [8]. In this rapid age of progress, a more novel approach is needed for a developing country like Pakistan.

With more than 5 billion worldwide mobile phone subscribers [9], mobile phone technology is rapidly progressing in the field of medicine especially for Short Message Service based interventions. In the last few years, Short Message Service (SMS) based interventions have displayed significant results for diseases like diabetes mellitus, HIV, and tuberculosis in both developed and developing countries [10, 11]. This form of intervention has proven to be cheap, accessible, and an easy way of communication [12].

Mobile phone use has risen remarkably over the last decade in Pakistan. Since 2004, the number of mobile phones in use in the country has increased from 5 million to 130 million, with almost every household in the country now having at least one mobile phone [13]. Even a normal SMS costs less than US$0.01 to send, and rates are even lower for computerized SMS packages.

Keeping our current study domain in mind, which has had a limited assessment in this regard in the past, we aim to assess the acceptability of SMS based interventions towards becoming future voluntary blood donors among medical university students of Karachi, Pakistan, so that a foundation may be laid in solving the solution of shortfall in a low income country like Pakistan. The secondary aim was to sensitize the medical student population regarding donating blood.

## 2. Methodology

### 2.1. Ethics Statement

The study has been approved by the Ethical Review Board of Dow University of Health Sciences. Each participant gave a written informed consent and all their information was kept confidential.

### 2.2. Sample Size

Using an estimated response distribution of 50%, as in previous studies [7, 14] conducted in other countries and a confidence interval of 95%, the sample size of this study was calculated to be a total of 350 medical students. A total of 432 participants were approached to participate in our study. 54 students were excluded for not meeting the respective inclusion criteria while 28 students declined to participate in the study.

### 2.3. Data Collection Process

The study was conducted amongst the two largest public sector medical universities of Karachi: Dow Medical College and Jinnah-Sindh Medical University from September 1, 2013, to September 30, 2013. Selection of these universities was based on the criteria of highest number of medical students studying in a single medical university in Karachi.

Coauthors of this study, who were also the data collectors, approached the participants at the study site. Limited availability of clinical years due to ward rotations and distributed schedules of nonclinical years made us choose nonprobability convenient sampling technique for this study. Each participant was initially assessed for eligibility criteria: participant should be a medical student currently enrolled in a 5-year MBBS program at his/her university with an age of 18 or above and should not be a registered voluntary blood donor. Any participant that failed to meet all three criteria was excluded from the study.

Data collectors informed each participant of the study topic and its implications. Participants agreeing to take part gave a written informed consent. Data collectors then administered the questionnaire to each participant using an interview based format. Each data collector verbally read out close ended questions from the questionnaire to the participants in a private and secluded place and recorded their answer on the questionnaire. An average of 5–10 mins was taken by a data collector to interview one participant. At the end, each participant was thanked and assured of complete confidentiality.

### 2.4. Questionnaire

Using template questions of Giri and Phalke's [7] questionnaire, we modified and designed a self-made questionnaire based on Pakistani blood bank settings for our study. The questionnaire was divided into four different sections.

- *General Blood Donation Knowledge*. This section assessed the familiarity of medical students regarding blood donations and working of blood banks in Pakistan.
- *Personal Blood Donation Attitude/Practices*. This section assessed personal approach of each medical student regarding previously donated blood, reasons of donating blood/not donating blood, fear of donating blood, and whether they would like to donate blood anytime in the future.
- *Implication of Being a Volunteer Blood Donor*. This section assessed the implications of medical students on becoming volunteer blood donors in Pakistan.
- *Short Message Service Intervention*. This section evaluated the baseline characteristics of Short Message Service and acceptability of participants towards any Short Message Service interventional studies to become voluntary blood donors in the future.

Close ended questions comprised the majority and important part of the questionnaire with multiple choice answers (yes, no, and maybe).

### 2.5. Statistical Analysis

All the data was recorded in a structured compilation sheet and analyzed through application of statistical tools of analysis. A descriptive statistical analysis was carried out on SPSS 16 software with mean, mode, and standard deviations calculated.

## 3. Results

350 participants, having a mean age of 21.47 ± 1.36, were included in our study with 30.6% (107/350) being males and 69.4% (243/350) being females.

The first component of our questionnaire dealt with knowledge of Pakistani blood bank working and blood donations (Table 1). 95.1% (333/350) participants were familiar about the compatibility of different blood transfusions while 90.3% (316/350) participants were aware of screening by blood banks. 62.9% (220/350) participants knew the right time permitted to redonate blood in Pakistan whereas 62.6% (219/350) participants knew that 50 kg was the borderline weight criterion for donating blood in Pakistan. Only 36.9% (129/350) participants knew that 450 mL blood is donated by a volunteer in Pakistan while 36% (126/350) participants did not have any idea. Similarly, knowledge of discarding unutilized blood products was low as well with 38.9% (136/350) participants having no clue and 34.2% (230/350) giving a wrong answer.

Our second component dealt with attitude and personal practices among the participants regarding donating blood. 93.4% (327/350) participants agreed that donating blood was healthy. A large number of the participants, 74% (259/350), had never donated blood in their life. Assessment of reasons for not donating blood found that 47.6% (123/259) did not fit blood donation criteria followed by 24.7% (64/259) who did not feel like donating. The rest of the participants never thought of donating.

Genderwise, 206/243 (84.8%) females did not donate blood in comparison to 53/107 (49.5%) males. The majority of the females, 53.3% (110/206), did not donate blood as they did not completely fit the blood donation criteria while 52.8% (28/53) of the males did not donate blood as they did not feel like donating. However, 60% (155/259) of the participants agreed to become voluntary blood donors through any sort of intervention.

Only 26% (91/350) participants were first time donors. Assessment of reasons among the first time donors found that 79.1% (72/91) had donated as replacement donors with the rest of them (19/91) as paid for donors. Furthermore, 87.9% (80/91) of these participants agreed to become regular voluntary blood donors if given an initiative, reminder, or boost.

Fear of donating blood was kept as a separate entity in our study. 68.6% (240/350) participants had no fear of donating blood while 31.4% (110/350) did have a fear. Evaluation of the reasons for fear among these 110 participants found that 36.36% (40/110) of the participants had a fear of needle prick followed by 33.6% (37/103) participants fearing that they would feel weak.

Genderwise, 71% (78/110) of the females had a fear of donating blood in comparison to 29% (32/110) of the males. Needle prick was the major fear factor for 38.4% (30/78) females not donating blood. 31.25% (10/32) of the males had a fear of needle prick with 31.25% (10/32) males also having a fear of feeling weak after donating. However, 88.3% (91/110) participants agreed to take part in an intervention to help remove the fear and become voluntary blood donors.

The third component of our study dealt with implications of knowing and becoming a voluntary blood donor in Pakistan (Table 2).

The last and most important component of our study dealt with acceptability and baseline characteristics in taking part in Short Message Service based interventions. Among baseline characteristics, 98.9% (346/350) participants had a cell phone and 96.9% (339/350) were comfortable using SMS (Table 3).

65.7% (230/350) of the total participants agreed to take part in SMS based behavioral interventions to become voluntary blood donors. The intervention could be related to removal of fear, promoting knowledge, or any other reason that may help the individual to donate blood voluntarily. 69.7% (244/350) of the participants gave a desirable answer that SMS reminders can remind and encourage them to donate blood more often. However, 40% (140/350) of the participants wanted an incentive (cash) to take part in such interventions to become future voluntary blood donors (Table 4).

## 4. Discussion

Over the last few decades, knowledge, attitudes, and decision making practices with regard to voluntary blood donation have been assessed in order to develop a better understanding of the process, so that donation efficiency, safety, retention, collection numbers, and donor pool diversity may be effectively regulated. In our current setup, results of our study act as pivotal tool in bringing about further improvements in future blood donation practices.

While knowledge of blood bank working and practices is not a major determinant in donor motivation [15], knowledge of donating blood among donors is an essential aspect for donor recruitment, especially volunteer donors [16]. The literature reveals that medical students are justifiably expected to have better knowledge of both blood donations and blood banks than other groups in society [15]. Our study reported medical students having an overall mixed approach on both aspects. Parts like screening and blood group compatibility had better results while amount of blood donated and discarding blood products showed low results. Overall, the results were below par in comparison to studies conducted in Greece and India [17, 18] but conformed more towards Alam and El Din Masalmeh's study in Saudi Arabia [19]. It is, however, important to note that all three aforementioned studies were done amongst the general population. Even Sabu et al.'s study on Indian medical students concluded much better results [20]. Such poor knowledge among Pakistani medical students acts as a major hindrance for voluntary blood donation.

93.4% of the participants deemed blood donation to be a healthy and positive practice, but only 26% students had actually ever donated blood. 15.2% of the Pakistani medical students in Siddiqui et al.'s study and 23% of the Pakistani medical students in Khan et al.'s study reported the same practice [14, 21]. Almost all medical students in both studies were also aware that donating blood was healthy. Similar studies conducted upon university students of other developing countries like Thailand, Iran, and India have also revealed similarly low donor percentages [1, 20, 22]. Shah et al.'s decade old behavioral study on Dhaka University students showed corresponding outcomes to our study as well [23]. This goes on to prove that while technology is soaring sky high with each passing day, behavioral patterns continue to remain stagnated, therefore highlighting a dire need for effective and motivational donor recruitment programs.

Careful assessment of factors which hinder a process is a key player in reaching a solution and formed a substantial portion of our study results. Among the nondonor respondents, the majority did not fit the blood donation criteria, comprising mostly females. The ratio of female students studying in Pakistani Medical Universities is very high as compared to male students. A similar finding was suggested by Hosain et al. [24]. Male participants, who did not donate, admitted that they did not feel like donating, reflecting a clear sense of nonchalance and lack of stimulation as compared to their female counterparts who wanted to donate but were not eligible to do so. These factors exhibit a high likeability to previous studies in Pakistan and other developing countries [21, 25, 26]. However, one South Indian study showed no statistical difference between gender and attitude toward donating blood [20].

The literature review has also explicitly unveiled fear as a colossal reason which hinders all types of potential donors from donating blood. Out of the 31.4% respondents who nurtured a fear, the majority hailed from amidst the female gender. Reasons of fear elicited were risk of catching infection and weakness after donating. However, fear of needle prick was the most popular response, comparable to Siddiqui et al.' study [14] as well as other studies on medical students [20, 25]. Much of this fear is reported to have escalated ever since HIV took over the global health scene by storm, sowing the seeds for apprehension among the already reluctant populace.

But despite low percentages reported in knowledge, attitude, and practices of our study and other studies in Pakistan, Siddiqui et al. suggested that medical students in Pakistan still comprise the major portion of regular voluntary donors and are more likely to be driven by altruism and humanitarian causes as compared to other groups in the donor pool [14]. This is clearly true, as 60% of nondonor respondents and 87.9% of first time donors in our study were willing to become regular voluntary blood donors through an intervention. Such health interventions for Pakistan will depend on accessibility, resourcefulness, cost, and time frame.

The dramatic rise of cellphone and SMS usage in Pakistan can be a solution to this problem. Baseline information of mobile phones in our study found that 98.9% participants had a cell phone and 96.9% were comfortable using SMS. These baseline results correlate with the information released by the Pakistan Telecommunication Authority (PTA). To date, almost every individual in Pakistan has at least one cellphone [13]. Furthermore, in 2011, an average of 250 daily texts was sent by an individual in Pakistan [13]. Such statistics prove that mobile phone usage in Pakistan has penetrated all levels of society, especially the lower class. A foundation to potentiate many other different types of SMS based health interventions in Pakistan can be based on this data.

A behavioral intervention is defined as improving or modifying a behavior for a positive impact. In our study, SMS behavioral interventions would aim at helping remove fear or improving knowledge by removing any taboo or misconception via SMS. 65.7% of the participants agreed to be part of such SMS based behavioral interventions indicating a positive attitude. Gombachika and Monawe's study among the general population of Malawi also indicated a positive attitude towards SMS based behavioral interventions [27]. Low lying, cheap, and accessible technology of mobile phones can easily be used by developing countries like Pakistan and Malawi to initiate SMS based interventions in improving blood donations.

SMS reminders for blood donation are also currently in use in many blood banks of developing countries including India [28]. 69.7% respondents of our study were affirmative in receiving SMS blood donation reminders. Fung et al.'s study stated better attendance rates amongst American donors who received SMS reminders, hence proving the effectiveness of SMS reminders [29]. SMS reminders can tackle the “nobody told me to donate” respondents and first time donors by continuous encouragement to them to donate. Blood banks can be made responsible for sending tailored SMS to the people and in turn use this data in developing a registry of voluntary blood donors that currently does not exist in Pakistan.

There are several limitations to our study. First of all, our study was limited to only a certain population that could read SMS. SMS based interventions will only work among those who can read SMS. This will rule out a large major portion of the population that is illiterate. Secondly, these SMS based interventions may become gender biased towards males. Females in Pakistan mostly do not donate blood due to blood donation criteria that cannot be changed by an intervention.

## 5. Conclusion

There is still major workup needed to create more awareness about blood donation among medical students keeping in mind their future professional link to it. We recommend inclusion of such awareness programs as part of curriculum of medical students. However, pertaining to attitude and practices, there are positive outcomes with high acceptability among medical students to take part in SMS based interventions to become voluntary blood donors. If proper authorities, especially blood banks, work on these interventions, a larger pool of voluntary blood donors in Pakistan can be built.

## Figures and Tables

**Table 1:** Knowledge of Pakistani blood banks and blood donations.

Question	Correct answer, *n* (%)	Wrong answer, *n* (%)	Did not know, *n* (%)
Weight criterion permitted in Pakistan	219 (62.6)	93 (26.6)	38 (10.8)
Compatibility of blood Transfusions	333 (95.1)	17 (4.1)	—
Screening by blood banks	316 (90.3)	34 (9.7)	—
Amount of donated blood permitted in Pakistan	129 (36.9)	95 (27.2)	126 (36)
Maximum components from a single donation	149 (42.6)	140 (40)	61 (17.4)
Number of days for discarding unutilized blood products in Pakistan	94 (26.9)	230 (65.7)	26 (7.4)
Time duration to redonate blood in Pakistan	220 (62.9)	110 (31.4)	20 (5.7)
Type of blood component commonly utilized in Pakistan	225 (64.3)	107 (30.5)	18 (5.2)

**Table 2:** Implications of knowing and becoming a voluntary blood donor.

Implications	Yes *n* (%)	No *n* (%)
Definition of voluntary blood donor	329 (94)	21 (6)
Asking family relatives/replacement donors to donate blood	317 (90.6)	33 (9.4)
Blood banks pay voluntary blood donors in Pakistan	265 (75.7)	85 (24.3)
Incentives should be given to voluntary blood donors in Pakistan	187 (53.4)	163 (46.6)
Maintaining a registry by blood banks of voluntary blood donors	328 (96.6)	12 (3.4)

**Table 3:** Baseline characteristics of participants for Short Message Service intervention.

Question	Answer (*n*)
Having a cell phone	(i) Yes = 346 (ii) No = 4 Total = **350**

Comfortable using SMS	(i) Yes = 339 (ii) No = 11 Total = **350**

Time of day comfortable receiving SMS	(i) Morning = 27 (ii) Afternoon = 32 (iii) Evening = 69 (iv) Night = 42 (v) Anytime = 180 Total = **350**

Language you wish to receive SMS	(i) English = 228 (ii) Urdu = 30 (iii) Roman Urdu = 92 Total = **350**

**Table 4:** Acceptability of taking part in SMS based interventions.

	Yes *n* (%)	No *n* (%)
Behavioral intervention	230 (65.7)	120 (34.3)
Blood donation reminder intervention	244 (69.7)	106 (30.3)
Want an incentive to take part	140 (40)	210 (60)
